# Main Active Components and Cell Cycle Regulation Mechanism of Astragali Radix and Angelicae Sinensis Radix in the Treatment of Ox-LDL-Induced HUVECs Injury and Inhibition of Their Cell Cycle

**DOI:** 10.1155/2021/8087183

**Published:** 2021-08-21

**Authors:** Cai-Xia Liu, Ying-Zi Tan, Chang-Qing Deng

**Affiliations:** ^1^School of Integrated Chinese and Western Medicine, Hunan University of Chinese Medicine, Changsha 410208, China; ^2^Medical School, Hunan University of Chinese Medicine, Changsha 410208, China

## Abstract

To explore the main active components and effects of cell cycle regulation mechanism of Astragali radix (AR) and Angelicae sinensis radix (ASR) on oxidative damage in vascular endothelial cells, a model of oxidative damage in human umbilical vein endothelial cells (HUVECs) induced by oxidized low-density lipoprotein (ox-LDL) treatment was developed. Based on the “knock-out/knock-in” model of the target component, cell viability, intracellular reactive oxygen species (ROS), and lactate dehydrogenase (LDH) leakage were assessed by Cell Counting Kit-8 assay, fluorescent probe 2,7-dichlorodihydrofluorescein diacetate (DCFH-DA), and colorimetric assay, respectively, to evaluate the protective effect of the active components of AR and ASR (astragaloside IV (AS IV), astragaloside I (AS I), formononetin (FRM), calycosin (CAL), calycosin-7-O-*β*-D glucoside (CLG), and ferulic acid (FRA)) against oxidative damage. The cell cycle and expression of genes encoding cyclins and cyclin-dependent kinases (CDKs) were observed using flow cytometry and quantitative real-time polymerase chain reaction. The results showed that the combination of active components (ACC) significantly inhibited the decrease in cell viability as well as the increase in ROS and LDH release in HUVECs induced by ox-LDL treatment. AS IV and FRM promoted the proliferation of HUVECs but the proliferation index was decreased in the AS I and FRA groups; this inhibitory effect was counteracted by the ACC. The ACC reduced and increased the proportion of positive cells in G1 and S phases, respectively, followed by the upregulation of cyclin A (*CCNA*), cyclin E (*CCNE*), and *CDK2* mRNA expression and downregulation of cyclin B (*CCNB*), cyclin D1 (*CCND1*), *CDK1*, *CDK4*, and *CDK6* mRNA expression, which significantly mitigated inhibition of HUVECs proliferation induced by ox-LDL treatment. Taken together, AS IV, AS I, FRM, CAL, CLG, and FRA were the primary pharmacodynamic substances of AR and ASR that alleviated oxidative injury in HUVECs. ACC mitigated the upregulation of intracellular ROS levels and LDH release induced by ox-LDL treatment, which promoted the cell cycle procession of HUVECs by regulating the expression of genes encoding cyclins and CDKs and thus preventing oxidative damage in HUVECs.

## 1. Introduction

Atherosclerosis (AS) is a chronic immune inflammatory disease characterized by abnormal lipid deposition on the arterial walls that causes various cardiovascular and cerebrovascular disorders, and AS is a common cause of human death and disability [1]. Cardiovascular diseases, such as AS, hypertension, and heart failure, contribute to an increase in the morbidity and mortality rates worldwide [2]. Although the etiology of cardiovascular disease is diverse, oxidative stress is a common underlying cause [2]. Vascular endothelial cells (VECs) not only act as a protective barrier between the blood and vascular wall but are also the primary targets of various environmental factors, such as hemodynamics, inflammatory cytokines, and oxidative stress. VECs dysfunction induced by oxidative stress is thought to be the initial stage and the essential pathological cause of AS [3]. Oxidative stress is associated with cell cycle arrest and apoptosis by inducing lipid peroxidation, cell membrane damage, DNA damage, and inflammatory factor release [2]. Accumulation of oxidized low-density lipoprotein (ox-LDL) induces oxidative stress and vascular endothelial dysfunction, thus promoting lipid accumulation in the subendothelial layer, atherosclerosis, and vascular stenosis [4]. Therefore, understanding the mechanism underlying vascular endothelial dysfunction induced by ox-LDL treatment and identifying potential drugs that can be used to protect VECs from oxidative damage may be effective methods for preventing and treating cardiovascular diseases such as AS.

Astragali radix (AR) and Angelicae sinensis radix (ASR) are classic pairs of drugs that invigorate qi and promote blood circulation, and either of them can protect VECs from oxidative damage due to its antioxidant properties. The compatibility of AR with ASR positively affects the proliferation and tubulogenic capacity of endothelial progenitor cells, increases the survival of VECs, and contributes to the protection of the cardiovascular system by inhibiting the apoptosis of endothelial progenitor cells induced by ox-LDL treatment [5, 6]. A previous study found that the compatibility of AR and ASR could alleviate vascular endothelial injury; the main active components detected in the plasma after the oral administration of Danggui Buxue Tang decoction were astragaloside IV (AS IV), calycosin (CAL), calycosin-7-O-*β*-D glucoside (CLG), astragaloside I (AS I), formononetin (FRM), and ferulic acid (FRA) [6, 7], and these components may protect the vascular endothelium. However, the pharmacological effects of these active components on oxidative damage in VECs remain unknown. In addition, it is not known whether a combination of active components differs from that of a single active component. Moreover, the mechanism of action of these active components in oxidative injury remains to be elucidated. Based on the model of oxidative stress in HUVECs induced by ox-LDL treatment, the “knock-out/knock-in” model of the target component [8] was developed to study the effects of each of the six main active components of AR and ASR and their effects when used in combination on the oxidative injury and cell cycle of HUVECs.

## 2. Materials and Methods

### 2.1. Reagents

The reference substances of the five active components of AR, including AS IV (Lot No. A0070), AS I (Lot No. A0071), FRM (Lot No. A0232), CAL (Lot No. A0514), and CLG (Lot No. A0515), and the reference substance of the active component of ASR, FRA (Lot No. A0050), were purchased from Chengdu Must Bio-Technology Co., Ltd (Chengdu, China). The purity of all reference substances was greater than 98%. These active components were dissolved in DMSO-DMEM, and the final concentration of DMSO was less than or equal to 0.1%. We obtained ox-LDL from Guangzhou Yiyuan Biotechnology Co., Ltd (Guangzhou, China).

### 2.2. Cell Culture and Administration

Immortalized human umbilical vein endothelial cells (HUVECs) used in this study, which expressed factor VIII-related antigen (VIIIR: Ag), were purchased from Shanghai Zhongqiao Xinzhou Biotechnology Co., Ltd. (Lot No. 19195, Shanghai, China). HUVECs were cultured in DMEM (Gibco, Carlsbad, CA, USA) supplemented with 10% fetal bovine serum (Gibco) and penicillin-streptomycin (100 U/mL media; Sigma-Aldrich, St. Louis, MO, USA) in a humidified atmosphere containing 5% CO_2_ at 37°C. These cells were referred to as HUVECs in all subsequent experiments.

With reference to our previous study [6, 9], the intermediate dose of these active components was selected, (AS IV (20 *μ*g/ml), AS I (7.5 *μ*g/ml), FRM (12.5 *μ*g/ml), CAL (10 *μ*g/ml), CLG (12.5 *μ*g/ml), and FRA (18 *μ*g/ml)), and six dosage groups (50% increment/decrement) were designed, respectively. Then, we detected the cell viability of HUVECs using the uniform design method and obtained the linear regression equation, so as to determine the compatible concentration of six active components. Besides, our team had detected the nontoxic concentration of six active components to HUVECs injury and the cell viability of HUVECs affected by the compatible concentration of all active components was less than 20% in this study.

The cells were grouped as follows: control group (control); ox-LDL group (model); active component groups (AS IV (2.5 *μ*g/ml), AS I (30 *μ*g/ml), FRM (1.5625 *μ*g/ml), CAL (1.25 *μ*g/ml), CLG (50 *μ*g/ml), and FRA (2.25*μ*g/ml)); active component knock-out groups (AS IV (−), AS I (−), FRM (−), CAL (−), CLG (−), and FRA (−)); and active component knock-in groups (active component combination, ACC). In the “knock-out/knock-in” model of the target component, the compatibility of all active components was considered (knock-in group), while one component was removed (knock-out group). AS IV was used as an example to introduce the knock-out and knock-in modes of these active components: (i) AS IV; (ii) AS IV knock-out/(−) group: AS I, FRM, CAL, CLG, and FRA; and (iii) AS IV knock-in/active component combination group (ACC): AS IV, AS I, FRM, CAL, CLG, and FRA. To detect the possible protective effects of the combination of AR and ASR against ox-LDL, HUVECs were treated with active components for 2 h prior to the ox-LDL challenge and then stimulated with 200 *μ*g/ml of ox-LDL for 24 h in the subsequent experiments.

### 2.3. Cell Viability Assay

The effects of the main active components of AR and ASR (AS IV, AS I, FRM, CAL, CLG, and FRA) on cell viability were assessed using the Cell Counting Kit (CCK-8) assay. Briefly, the cells were counted, adjusted, and seeded onto 96-well plates, with each group in the plate containing six copies of cells. The cells were either left untreated or treated with the main active components in the presence or absence of ox-LDL. According to the manufacturer's protocol, CCK-8 solution (10 *μ*l) was added to each well, and the cell culture plate was incubated for 1–4 h at 37°C. The absorbance was measured at 450 nm using a microplate reader (Thermo Fisher Scientific, Waltham, MA, USA).

### 2.4. Measurement of Intracellular Reactive Oxygen Species Content

In accordance with previous studies [10], intracellular reactive oxygen species (ROS) levels were measured using a biochemical assay kit (Elabscience Biotechnology Co., Ltd., Wuhan, China). After incubation with 10 *μ*mol/l of 2′,7′-dichlorodihydrofluorescein diacetate (DCFH-DA) for 30–60 min at 37°C in the dark, the cells in the logarithmic growth phase were washed with buffer solution three times. Next, the fluorescence intensity of 2′,7′-dichlorofluorescein (DCF) was monitored using a laser scanning microscope (Carl Zeiss, Jena, Germany) system and microplate reader (Thermo Fisher Scientific).

### 2.5. Cell Cycle Assay

Flow cytometry (FCM) was used to assess cell cycle distribution in HUVECs grown until 70%–80% confluency. HUVECs were either left untreated or treated with active components, with or without ox-LDL. The cells were harvested, washed with PBS, and fixed overnight. The fixed cells were washed, incubated with RNase A (100 *μ*g/ml, Sigma-Aldrich), stained with propidium iodide (PI, 50 *μ*g/ml; Sigma-Aldrich) in the dark, and analyzed by FCM. PI-free cells were used as negative controls. The proliferation index (Pi) was calculated as follows:

(1) $$\begin{array}{c} \text{Pi}=\frac{\left(S+G2/M\right)}{\left(G0/G1+S+G2/M\right)}\times 100\%. \end{array}$$

### 2.6. Lactate Dehydrogenase

As a biomarker of cell injury, lactate dehydrogenase (LDH) present in HUVECs is an intracellular enzyme that is released into the culture media upon cell damage. The media and lysates from HUVECs were collected, and the LDH content was determined using a microplate reader (Thermo Fisher Scientific) according to the specifications of the LDH assay kit (Beyotime Biotech Inc., Shanghai, China). The rate of LDH leakage was expressed as the ratio of LDH levels in the media to those in the supernatant and cell lysate.

### 2.7. Total RNA Isolation and Quantitative Real-Time Polymerase Chain Reaction (qRT-PCR) Analysis

Total RNA was extracted using an RNAiso Plus solution (TaKaRa, Kusatsu, Japan) following the manufacturer's protocol. Reverse transcription was performed using the RevertAid First Strand cDNA Synthesis Kit (Thermo Fisher Scientific). Subsequently, qRT-PCR was performed on an Applied Biosystems 1900 Analyzer (Applied Biosystems, Foster City, CA) using the SYBR Green I Real-Time PCR Kit (Bio-Rad, Hercules, CA). The primers were synthesized using the sequences listed in Table 1. The expression of the housekeeping gene, *GAPDH*, was used to normalize the expression of target genes with approximately equal amplification efficiency.

### 2.8. Statistical Analysis

All experiments were repeated at least three times. Statistical analyses were performed using SPSS 25.0. Data are presented as the mean ± SD and analyzed using one-way analysis of variance for multiple comparisons. Statistical significance was set at *P* < 0.05.

## 3. Results

### 3.1. ACC Inhibits the Decrease in HUVECs Viability Induced by Ox-LDL Treatment

**Figure 1:**
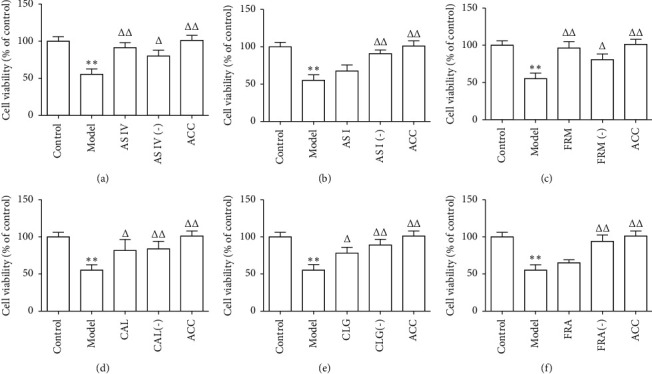
Six active components improved the viability of HUVECs treated with ox-LDL. Cell viability was determined using the Cell Counting Kit-8 (CCK-8) assay. Results are expressed as the mean ± standard deviation of independent experiments performed in triplicate and normalized to control (*n* = 4). AS IV: astragaloside IV; AS I: astragaloside I; FRM: formononetin; CAL: calycosin; CLG: calycosin-7-O-*β*-D glucoside; FRA: ferulic acid; active components (−): active component knock-out; ACC: active component combination. ^∗∗^*P* < 0.01 vs. control; ^Δ^*P* < 0.05, ^ΔΔ^*P* < 0.01 vs. model.

We detected the effect of the main active ingredients from AR and ASR on the viability of HUVECs using the CCK-8 assay. Ox-LDL treatment reduced HUVECs viability by approximately 50% (Figure 1). Compared with the model group, AS IV, FRM, CAL, and CLG significantly increased the viability of HUVECs. AS IV and FRM, which had better effects than CAL and CLG, increased the viability of HUVECs by 65.11% and 74.52%, respectively (Figures 1(a) and 1(c)). Either AS I or FRA, used as a single component, had no significant effects on cell viability (Figures 1(b) and 1(f)). All the active component knock-out groups effectively inhibited the decrease in HUVECs viability induced by ox-LDL treatment. The viability of HUVECs in the knock-in group (ACC) increased by 83.15% compared with the model group.

### 3.2. Influence of ACC on the Intracellular Levels of ROS in HUVECs Treated with Ox-LDL

**Figure 2:**
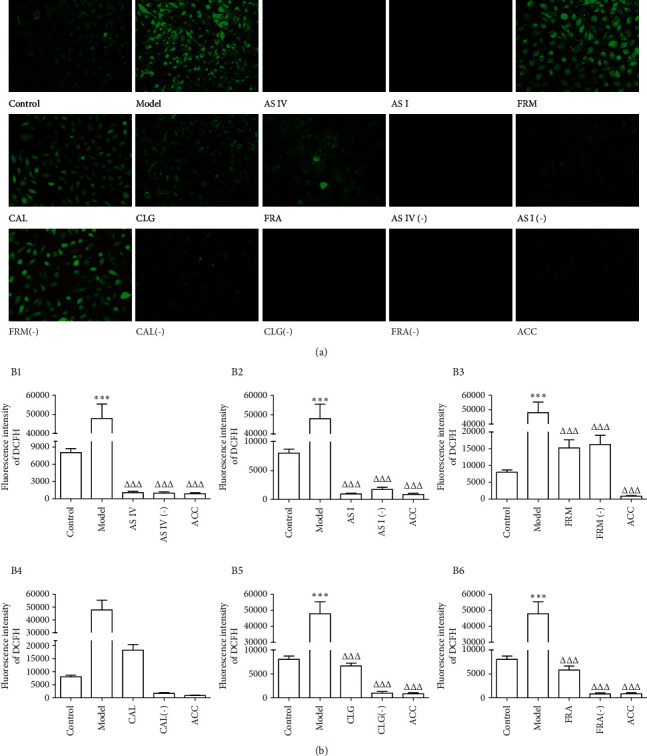
Effects of the six active components on intracellular ROS levels in HUVECs. The cells were labeled using DCFH-DA, and the fluorescence intensity of DCF was monitored with a laser scanning confocal microscope (*n* = 3). AS IV: astragaloside IV; AS I: astragaloside I; FRM: formononetin; CAL: calycosin; CLG: calycosin-7-O-*β*-D glucoside; FRA: ferulic acid; active components (−): active component knock-out; ACC: active components combination.

**Figure 3:**
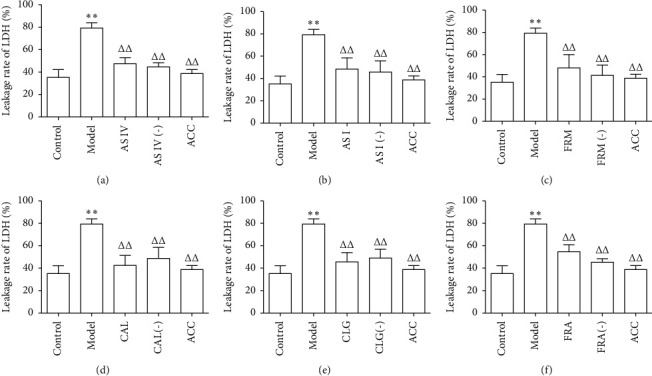
Six active components of AR and ASR decreased the leakage rate of LDH in HUVECs treated with ox-LDL. LDH release was assessed using an LDH assay kit (*n* = 5). AS IV: astragaloside IV; AS I: astragaloside I; FRM: formononetin; CAL: calycosin; CLG: calycosin-7-O-*β*-D glucoside; FRA: ferulic acid; active components (−): active component knock-out; ACC: active component combination. ^∗∗^*P* < 0.01 vs. control; ^ΔΔ^*P* < 0.01 vs. model.

Next, the level of ROS in HUVECs was measured using the fluorescent probe DCFH-DA. As shown in Figure 2, intracellular ROS levels in HUVECs treated with ox-LDL significantly increased, suggesting that ox-LDL could cause oxidative stress injury in HUVECs. Compared with the model group, the six active components of AR and ASR significantly reduced the level of ROS in HUVECs. AS IV and AS I reduced the fluorescence of DCFH in HUVECs by more than 95% (Figure 2(b); 1, 2). The intracellular ROS levels in the FRM and CAL groups decreased by 68.13% and 61.86%, respectively (Figure 2(b); 3, b4). CLG and FRA reduced ROS levels in HUVECs by approximately 85% (Figure 2(b); 5, b6). The combination of the six active components significantly inhibited the increase in ROS levels in HUVECs.

### 3.3. Effects of the Six Active Components on the Rate of LDH Leakage in HUVECs Treated with Ox-LDL

To evaluate cell injury, we measured the LDH leakage rate of HUVECs using a colorimetric assay. The results in Figure 3 show that ox-LDL caused a significant increase in LDH release in HUVECs, suggesting that ox-LDL-induced cell injury. The six active components of AR and ASR caused a marked decrease in the leakage rate of LDH and alleviated cell damage, while ACC had a synergistic effect. Among the active components, the effect of CAL was the strongest and reduced the leakage rate of LDH by approximately 50% (Figure 3(d)). The leakage rate of LDH in the group treated with FRM, whose effect was weak, was 31.12% less than that in the model group (Figure 3(c)).

### 3.4. Effect of Active Components of AR and ASR on the Proliferation of HUVECs

**Figure 4:**
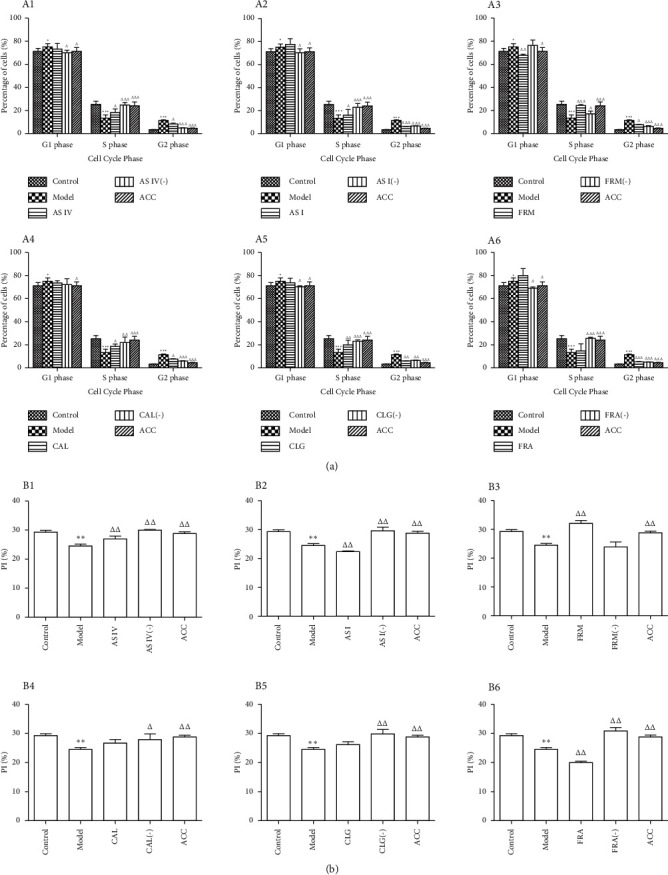
Influence of the six active components of AR and ASR on the cell cycle and Pi of HUVECs treated with ox-LDL. The cell cycle was analyzed by FCM, and the proliferation index (PI) was defined as the ratio of cells in the S and G2/M phases to cells in the G0/G1, S, and G2/M phases. Results are expressed as the mean ± SD of independent experiments performed in triplicate (*n* = 3). A1 to A6 represent the percentages of cells in each cell cycle phase. B1 to B6 represent the Pi of HUVECs. AS IV: astragaloside IV; AS I: astragaloside I; FRM: formononetin; CAL: calycosin; CLG: calycosin-7-O-*β*-D glucoside; FRA: ferulic acid; active components (−): active component knock-out; ACC: active component combination. ^∗∗^*P* < 0.01 vs. control; ^Δ^*P* < 0.05, ^ΔΔ^*P* < 0.01 vs. model ^ΔΔΔ^*P* < 0.01.

To explore the effect of the six active components of AR and ASR on the proliferation of HUVECs, we assessed the cell cycle of HUVECs using flow cytometry. Compared with the control group, the proportion of cells in the G1 phase in the model group increased, the proportion of cells in the S phase decreased, and the Pi of HUVECs significantly reduced, indicating that the proliferation of HUVECs was inhibited (Figure 4). FRM and AS IV were more efficient in promoting HUVECs proliferation (Figure 4; B1, B3); moreover, the effects of CAL and CLG on the proliferation of HUVECs were weaker than those of FRM and AS IV (Figure 4; B4, B5). AS I and FRA reduced the Pi of HUVECs and inhibited their proliferation (Figure 4; B2, B6).

### 3.5. Influence of the Six Active Components on the Expression of Genes Encoding Cyclins in HUVECs Treated with Ox-LDL

**Figure 5:**
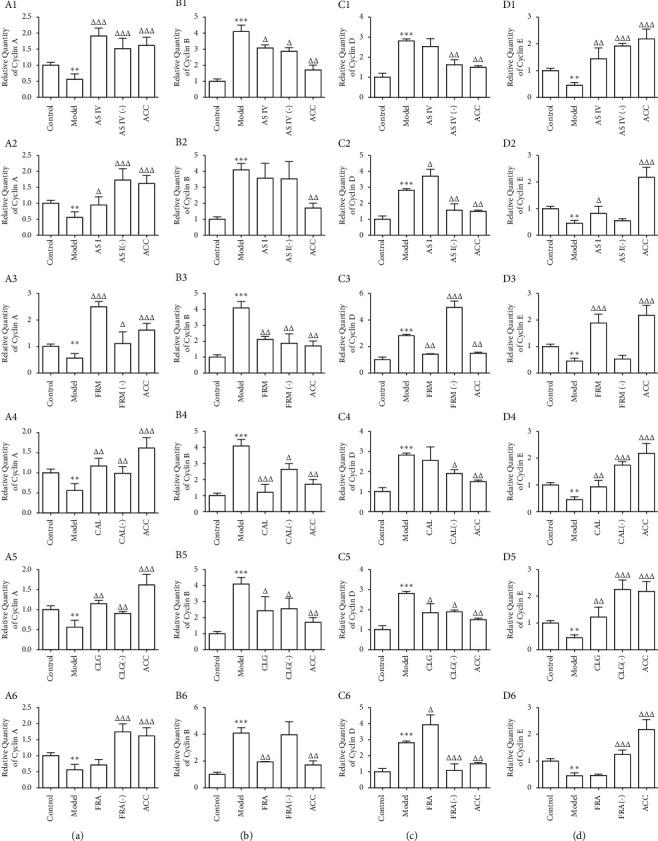
Expression of cyclins at the mRNA level in HUVECs was determined by qRT-PCR. Results represent the mean of three independent experiments (*n* = 3). A1 to A6 indicate the expression of cyclin A at the mRNA level. B1 to B6 represent the expression of cyclin B at the mRNA level. C1 to C6 show the expression of cyclin D at the mRNA level. D1 to D6 display the expression of cyclin E at the mRNA level. Results are expressed as the mean ± standard deviation of independent experiments performed in triplicate and normalized to control. AS IV: astragaloside IV; AS I: astragaloside I; FRM: formononetin; CAL: calycosin; CLG: calycosin-7-O-*β*-D glucoside; FRA: ferulic acid; active components (−): active component knock-out; ACC: active component combination. ^∗∗^*P* < 0.01 vs. control; ^Δ^*P* < 0.05, ^ΔΔ^*P* < 0.01, ^ΔΔΔ^*P* < 0.001 vs. model.

Treatment with the six active components of AR and ASR significantly influenced the expression of four cyclin-coding genes, including those encoding cyclin A (*CCNA*), cyclin B (*CCNB*), cyclin D1 (*CCND1*), and cyclin E (*CCNE*). Compared with the control group, the mRNA levels of genes encoding cyclin A and cyclin E decreased by 43.87% and 53.89% in the model group, but the mRNA levels of genes encoding cyclin B and cyclin D1 increased by 306.97% and 177.96%, respectively (Figure 5).

AS IV and CAL elevated the expression of *CCNA* and *CCNE* (Figure 5 A1, A4, D1, and D4) and reduced the expression of *CCNB* (Figure 5; B1, B4), but they had no noticeable influence on the expression of *CCND1* (Figure 5; C1, C4). The effects of FRM and CLG were characterized by increased levels of *CCNA* and *CCNE* (Figure 5; A3, A5, D3, and D5), and decreased levels of *CCNB* and *CCND* (Figures 5; B3, B5, C3, and C5). Compared with the model group, except for *CCNB*, the expression of *CCNA*, *CCND1*, and *CCNE* in the AS I group significantly increased (Figures 5; A2, B2, C2, and D2). FRA downregulated and upregulated the mRNA levels of the genes encoding *CCNB* and *CCND1*, respectively (Figures 5; B6, C6); however, this active component had no significant effects on the expression of *CCNA* and *CCNE* (Figures 5; A6, D6). The combination of the six active components reversed the transcription of the cyclin-coding genes induced by ox-LDL treatment; in addition, the six active components upregulated the expression of *CCNA* and *CCNE* and downregulated that of *CCNB* and *CCND1*.

### 3.6. Effect of the Six Active Components on the mRNA Expression of Genes Encoding Cyclin-Dependent Kinases in HUVECs Treated with Ox-LDL

**Figure 6:**
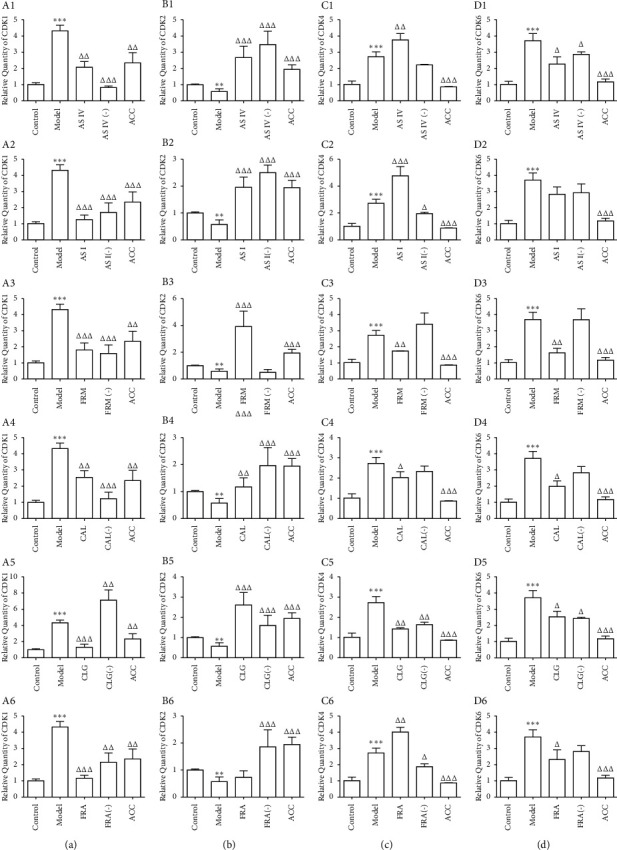
Effect of the six active components on the expression of CDKs at the mRNA level in HUVECs. The expression at the mRNA level was evaluated by qRT-PCR, and the data represent the average of three experimental results (*n* = 3). A1 to A6 represent the expression of CDK1 at the mRNA level. B1 to B6 display the expression of CDK2 at the mRNA level. C1 to C6 represent the expression of CDK4 at the mRNA level. D1 to D6 show the expression of CDK6 at the mRNA level. AS IV: astragaloside IV; AS I: astragaloside I; FRM: formononetin; CAL: calycosin; CLG: calycosin-7-O-*β*-D glucoside; FRA: ferulic acid; active components (−): active component knock-out; ACC: active component combination. ^∗∗^*P* < 0.01, ^∗∗∗^*P* < 0.001 vs. control; ^Δ^*P* < 0.05, ^ΔΔ^*P* < 0.01, ^ΔΔΔ^*P* < 0.001 vs. model.

To explore the mechanism of action of the main active components of AR and ASR on the expression of cyclins in HUVECs, we investigated the expression of four cyclin-dependent kinases (CDKs), including CDK1, CDK2, CDK4, and CDK6. The data in Figure 6 show that the expression of *CDK1*, *CDK4*, and *CDK6* in HUVECs in the ox-LDL group increased, while the expression of *CDK2* decreased.

Compared with the model group, AS IV significantly reduced the expression of *CDK1* and *CDK6* (Figure 6; A1, D1) and elevated the expression of *CDK2* and *CDK4* in HUVECs treated with ox-LDL (Figure 6; B1, C1). Similar to AS IV, AS I also inhibited the mRNA expression of *CDK1* (Figure 6; A2) and promoted the mRNA expression of *CDK2* and *CDK4* (Figure 6; B2, C2); however, AS I had no significant effect on *CDK6* mRNA levels (Figure 6; D2). The effects of FRM, CAL, and CLG were analogous; they downregulated the mRNA expression of *CDK1*, *CDK4*, and *CDK6* (Figures 6; A3-A5, C3-C5, D3-D5) and upregulated the mRNA expression of *CDK2* (Figures 6; B3–B5) in HUVECs. FRA, the active component of ASR, reduced the expression of *CDK1* and *CDK6* (Figures 6; A6, D6) and increased the expression of *CDK4* (Figure 6; C6), but it did not affect the mRNA levels of *CDK2* (Figure 6; B6).

## 4. Discussion

VECs, the monolayer squamous epithelial cells located on the inner surface of the blood vessels, act as a protective barrier between the blood and vascular wall. In addition, VECs play an essential role in maintaining the integrity of the vascular endothelium and homeostasis of the internal environment, and they participate in the regulation of oxidation-antioxidation, proinflammatory-anti-inflammatory functions, coagulation-anticoagulation, and vascular vasomotoricity [11]. VECs are challenged by various types of damaging stimuli during the development of AS, such as ox-LDL, leading to vascular endothelial injury [12]. As an important risk factor for the development of AS, ox-LDL causes dysfunction of the vascular endothelium by triggering the accumulation of lipids in the arterial walls, oxidative stress, mitochondrial dysfunction, permeability, and apoptosis of endothelial cells [13]. Therefore, exploring the mechanism and the effective antagonistic components of VEC damage induced by ox-LDL treatment may assist the development of a potential therapy for the prevention and treatment of AS.

An incomplete understanding of the molecular mechanisms involved in AS is one of the important factors that restrict the effective control of its progression, and oxidative stress is one of the main causes underlying the pathogenesis of this disease [14]. Oxidative stress has been recognized as the excessive production of highly active molecules, such as reactive nitrogen free radicals or ROS, which cause protein denaturation, lipid peroxidation of biomembranes, DNA damage, and apoptosis or cell death. Therefore, ROS are considered to be signaling molecules that lead to endothelial dysfunction during the development of AS. Ox-LDL plays an essential role in the early stages of AS by inhibiting cell proliferation, promoting inflammation, and inducing the apoptosis of endothelial cells [15, 16]. Excessive ROS levels can lead to the apoptosis or necrosis of endothelial cells by causing irreversible damage to the DNA, proteins, RNA, and other biological molecules. Previous studies have shown that ROS affect cell proliferation and differentiation by directly or indirectly regulating the transcription of genes encoding cell signaling-related factors [15]. LDH, which mediates cell survival, can be considered an indicator of the degree of cell membrane damage. Traditional Chinese medicine (TCM), which involves the use of natural products, has been recognized as a curative treatment for cardiovascular diseases such as AS [17]. In TCM, the combination of AR and ASR, which invigorates qi and activates blood circulation, strongly promoted tubulogenesis, induced the proliferation and migration of endothelial progenitor cells, and exerted anti-inflammatory and antioxidant effects on HUVECs that conferred cardiovascular protection [5]. In the present study, ox-LDL resulted in a decrease in cell viability, an increase in ROS content, and LDH release in HUVECs, suggesting the successful establishment of an oxidative damage model. The results showed that each of the six active components from AR and ASR significantly reduced the leakage rate of LDH and ROS levels; moreover, AS IV, FRM, CAL, and CLG, when used alone, markedly increased the activity of HUVECs. Furthermore, the combination of the six active components had synergistic effects, which mitigated oxidative damage in HUVECs by enhancing cell viability and reducing ROS content and LDH release.

**Figure 7:**
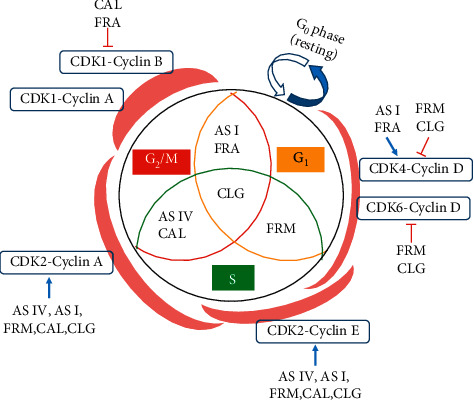
Schematic diagram showing the effects of the active components of AR and ASR on the cell cycle progression of HUVECs as well as the regulation of major cyclin-CDK complexes. AS IV: astragaloside IV; AS I: astragaloside I; FRM: formononetin; CAL: calycosin; CLG: calycosin-7-O-*β*-D glucoside; FRA: ferulic acid.

Studies have shown that administration of >50 *μ*g/ml of ox-LDL inhibits cell viability, proliferation, and even apoptosis [18]. Treatment with ox-LDL (200 *μ*g/ml), which led to an increase and a decrease in the proportion of cells in the G1 and S phases, respectively, reduced the Pi of HUVECs and inhibited the proliferation of the cells. The cell cycle process is driven by cyclins, which recognize and combine with CDKs, its catalytic chaperone [19]. The cyclin-CDK complex promotes the initiation of the G1 phase in cells and regulates the synthesis of DNA (S phase) to ensure the orderly completion of the cell cycle [19]. Based on the findings of previous studies on cyclins and CDKs [20], we summarized the effects of the active components on the cell cycle progression of HUVECs and the regulation of the levels of major combinations of cyclins and CDKs (Figure 7). CDK4 and CDK6 mainly bind to cyclin D1/D2/D3, and their expression increases from the G1 phase to the end of the mitosis phase [20, 21]. The binding targets of CDK2 are cyclin A and cyclin E, which participate in the transformation from G1 phase to S phase; CDK1 binds to cyclin A and cyclin B, promoting the cells to enter the mitosis phase from the G2 phase [19, 20]. In the present study, ox-LDL upregulated *CDK1*, *CDK4*, and *CDK6* mRNA expression; downregulated *CDK2* mRNA expression; increased the mRNA levels of genes encoding cyclin B, cyclin D1, and cyclin E; and decreased the mRNA levels of the cyclin A-coding gene in HUVECs, resulting in an increase and a decrease in the proportion of cells in the G1 and S phases, respectively. These events explain the mechanism through which ox-LDL inhibits the proliferation of HUVECs. Both AS IV and FRM significantly promoted HUVECs proliferation. Both components elevated the expression of *CDK2*, cyclin A, and cyclin E at the mRNA level and reduced the expression of *CDK1* and cyclin B at the mRNA levels; FRM downregulated the expression of *CDK4*, *CDK6*, and cyclin D1 at the mRNA level in HUVECs, but AS IV had no significant effect on the expression of the cyclin D1-coding gene. Interestingly, AS I and FRA, when used alone, decreased the Pi of HUVECs and inhibited their proliferation, but the combination of the six active components counteracted the inhibitory effect. The combination of the six active components increased the expression of *CDK2*, cyclin A, and cyclin E and decreased the expression of *CDK1*, *CDK4*, *CDK6*, cyclin B, and cyclin D1 at the mRNA level, thereby promoting the proliferation of HUVECs, which may be one of the mechanisms through which AR and ASR synergistically exert protective effects against oxidative damage in HUVECs treated with ox-LDL.

In summary, the combination of the six active components of AR and ASR inhibited the increase in ROS levels and LDH release and the decrease in HUVECs viability following ox-LDL treatment, indicating their antioxidant effects. AR and ASR also improved the proliferation of HUVECs by regulating the expression of CDKs and cyclins at the mRNA level; this result can serve as the basis for conducting further research on the cardiovascular-protective effects of AR and ASR and developing strategies for the prevention and treatment of AS.

However, due to the various compositions of TCM and the complex regulatory systems involved in oxidative damage and cell proliferation, it is unclear which of the aforementioned processes are linked to the active components. Whether other active components related to the compatibility of AR and ASR exist is also unknown. In addition, the existence of other interactions among the active components and cyclin/CDKs complexes is not known. Notably, it remains a challenge to combine the data obtained in the present study with those of in vivo studies to apply them in clinical practice and improve the prevention and treatment of cardiovascular diseases. To overcome these challenges, additional studies are required.

## Figures and Tables

**Table 1:** List of primers used in the qRT-PCR analysis.

Gene	Sequence of primers
GAPDH	Forward 5′-GAAGGTGAAGGTCGGAGTC-3′ Reverse 5′-GAAGATGGTGATGGGATTTC-3′
Cyclin A	Forward 5′-AGGCTAACCCCACTCTATGAATC-3′ Reverse 5′-TCTTGCCTTTGGTGGACTA-3′
Cyclin B	Forward 5′-CTTTGCACTTCCTTCGGAGA-3′ Reverse 5′-GTAGAGTTGGTGTCCATTCACC-3′
Cyclin D1	Forward 5′-CCTCGGTGTCCTACTTCAAATG-3′ Reverse 5′-CACTTCTGTTCCTCGCAGAC-3′
Cyclin E	Forward 5′-ACACCCTCTTCTGCAGCCTA-3′ Reverse 5′-ATCTCGTCCCCTGAACAAGC-3′
CDK1	Forward 5′-ACGCACCCCAACTACAACTC-3′ Reverse 5′-TCTCCTTAATGTCACGCACGA-3′
CDK2	Forward 5′-CCACCCCAATATTGTCAAGC-3′ Reverse 5′-AGTCGGCTAACTTGATGGAG-3′
CDK4	Forward 5′-GCTGCTGGAAATGCTGAC-3′ Reverse 5′-CACTCCATTGCTCACTCC-3′
CDK6	Forward 5′-GGACTTTCTTCATTCACACCG-3′ Reverse 5′-GACCACTGAGGTTAGGCCA-3′

## Data Availability

The data used to support the findings of this study are included within the article.
